# Logical model of telenursing program of a high complexity oncology care center^*^

**DOI:** 10.1590/1980-220X-REEUSP-2022-0067en

**Published:** 2022-07-25

**Authors:** Chrisna de Sousa Silva Mendes, Priscila Rangel de Souza, Andrea Rabelo, Adriana Marques da Silva, Maria Rita da Silva, Daniela Vivas dos Santos, Patrícia Coelho de Soárez

**Affiliations:** 1 Universidade de São Paulo, Faculdade de Medicina, São Paulo, Brazil.; 2 Instituto do Câncer do Estado de São Paulo, São Paulo, SP, Brazil.; 3 Oncologia D’Or, São Paulo, SP, Brazil.; 4 Universidade de São Paulo, Faculdade de Medicina, Departamento de Medicina Preventiva, São Paulo, SP, Brazil.

**Keywords:** Telenursing, Medical Oncology, Program Evaluation, Teleenfermería, Oncología Médica, Evaluación de Programas y Proyectos de Salud, Telenfermagem, Oncologia, Avaliação de Programas e Projetos de Saúde

## Abstract

**Objective:**

To develop the logical model of the *Alô Enfermeiro* program aiming at elucidating the existing structure, activities carried out, and expected results, allowing the program implementation systematic evaluation.

**Method:**

This is an evaluative study with a qualitative approach. The development of the logical model was based on systematic methodologies, constituted from the analysis of institutional documents, literature review, search for essential elements that supported the implementation of the program, and the participation of stakeholders for discussion and validation of the data obtained.

**Results:**

It was possible to define the macro problem that gave rise to the program, establish the definition of the Program *Alô Enfermeiro*, target audience, general and specific objectives, as well as to structure the necessary components, such as inputs and activities, indicating the expected results in the short, medium, and long term. The logical model allowed the identification of the *Alô Enfermeiro* Program evaluation question, directed to the evaluation of results.

**Conclusion:**

The logical model developed allowed the comprehension of the program structure, the interaction among the activities carried out and the expected results of the “*Alô Enfermeiro*”.

## RESUMO

**Objetivo:**

Desenvolver o modelo lógico do programa Alô Enfermeiro com o intuito de elucidar a estrutura existente, atividades realizadas e resultados esperados, possibilitando a avaliação sistemática da implementação do programa.

**Método:**

Trata-se de uma pesquisa avaliativa de abordagem qualitativa. O desenvolvimento do modelo lógico foi embasado em metodologias sistemáticas, constituídas a partir da análise dos documentos institucionais, revisão da literatura, busca por elementos essenciais que fundamentaram a implementação do programa, e a participação dos *stakeholders* para discussão e validação dos dados obtidos.

**Resultados:**

Foi possível definir o macroproblema que deu origem ao programa, estabelecer a definição do Programa Alô Enfermeiro, público-alvo, objetivos geral e específicos, além de estruturar os componentes necessários, como insumos e atividades, indicando os resultados esperados em curto, médio e longo prazo. O modelo lógico permitiu a identificação da pergunta avaliativa do Programa Alô Enfermeiro, direcionada à avaliação de resultados.

**Conclusão:**

O modelo lógico desenvolvido possibilitou a compreensão da estrutura do programa, da interação entre as atividades realizadas e os resultados esperados do “Alô Enfermeiro”.

## RESUMEN

**Objetivo:**

Desarrollar el modelo lógico del programa “Alô Enfermeiro” con el objetivo de elucidar la estructura existente, actividades realizadas y resultados esperados para que se pueda hacer la evaluación sistemática de la implementación del programa.

**Método:**

Se trata de una investigación evaluativa de abordaje cualitativo. El desarrollo del modelo lógico basó en metodologías sistemáticas, constituidas a partir del análisis de los documentos institucionales, revisión de la literatura, busca por elementos esenciales que fundamentaron la implementación del programa, y la participación de los *stakeholders* para debate y validez de los datos obtenidos.

**Resultados:**

Fue posible definir el macro problema que originó el programa, establecer la definición del Programa “Alô Enfermeiro”, público destinatario, objetivo general y específicos, además de estructurar los componentes necesarios como, por ejemplo, insumos y actividades, indicando los resultados esperados a corto, medio y largo plazo. El modelo lógico permitió la identificación de la pregunta evaluativa del Programa “Alô Enfermeiro” direccionada a la evaluación de resultados.

**Conclusión:**

El modelo lógico desarrollado facilitó la comprensión de la estructura del programa, de la interacción entre las actividades realizadas y los resultados esperados del “Alô Enfermeiro”.

## INTRODUCTION

Cancer patients’ treatment routine takes place mainly on an outpatient basis, which, despite the benefits, represents an important challenge for the health team, since treatment- and non-treatment-related complications can occur outside the hospital environment^(1)^.

Telenursing (TN), defined by the International Council of Nurses (ICN) as the practice of care, educational, managerial, and research nursing carried out at a distance, through electronic means^(2,3)^, has shown significant benefits^(4,5)^, and has been cited as a resource for the management of toxicities and symptoms in cancer patients^(6,7)^.

At the Cancer Institute of the State of São Paulo (ICESP), the implementation of a 24-hour care TN program was required. Currently, this program is called “*Alô Enfermeiro*” (*PAE*). *PAE* operations began in 2006 with the Oncology nursing team at the Radiology Institute (InRAD), and were transferred after the inauguration of ICESP. The volume of daily consultations has grown progressively over the years. In 2021, *PAE* made more than 54 thousand receptive consultations, that is, the ones in which the patient/companion contacts the program center, and about 30,000 active consultations, that is, those that the nurse contacts the patient/companion.

A program evaluation allows the monitoring of its progress towards goals, the identification of necessary modifications, and the judgment of success when reaching the results^(8)^. To design an evaluation plan, it is important to understand the program structure and to correlate available resources and interventions with desired outcomes.

The logical model (LM) has been widely used for structuring complex health intervention programs^(9–11)^, and pointed out as a tool that can guide the development, implementation, and evaluation of a given program^(12)^ .

The LM is defined as a graphical representation that allows the broad visualization of program components, to support the decision-making of managers in charge of the improvement and achievement improvement of results of the intervention in question^(10,13,14)^ .

The LM can help conceptualize complexity by describing the intervention components and the relations among them, making the “theory of change” and assumptions about causal pathways between the intervention and various outcomes explicit, and displaying the interactions between the intervention and the system in which it is implemented. It provides a framework to support the assessment, help interpret the results, as well as identify new evaluative questions and areas where more evidence is required^(15)^.

The participation of stakeholders in the LM construction is pivotal to the obtainment of different points of view about each topic discussed, and based on them, reach the consensus of those involved. In general, the stakeholders are from three groups: the individuals involved in the operation; those who are directly affected by the program; and those who will use the assessment results. The interaction and agreement among stakeholders is a key factor for the LM to generate assessments that actually represent the needs of the team and its beneficiaries^(8)^.

The literature on the development of the LM of a 24-hour care TN program for cancer patients in the Brazilian public health system is scarce.

This study aims at developing the logical model of the *PAE* to elucidate the existing structure, activities carried out, and expected results, allowing the program implementation systematic evaluation.

## METHOD

### Design of Study

This is an evaluative research, with a qualitative approach, to analyze the implementation of *PAE* at *ICESP*. The process of implementing *PAE* since 2006 was considered, time when operations took place at InRAD. Then, the program was transferred, formally structured, and implemented at ICESP in 2008. The construction of the LM constituted the first step in the analysis of the *PAE* implementation process.

The development of the LM followed the recommendations of the Institute of Applied Economic Research (*IPEA*)^(16)^, Centers for Disease Control and Prevention (CDC)^(8)^ and the INTEGRATE-HTA Project^(15)^, with some adaptations to avoid impacting the stakeholders’ work routine.

### Local

The present study was carried out at ICESP, referred to as a high complexity oncology center (CACON), with exclusive care for the Brazilian Public Health System (SUS) oncology patients, located in the city of São Paulo, Brazil, and certified by important institutions that value patient safety and quality of the care provided, such as the Joint Commission International (JCI).

### Data Collection

**Figure 1.**
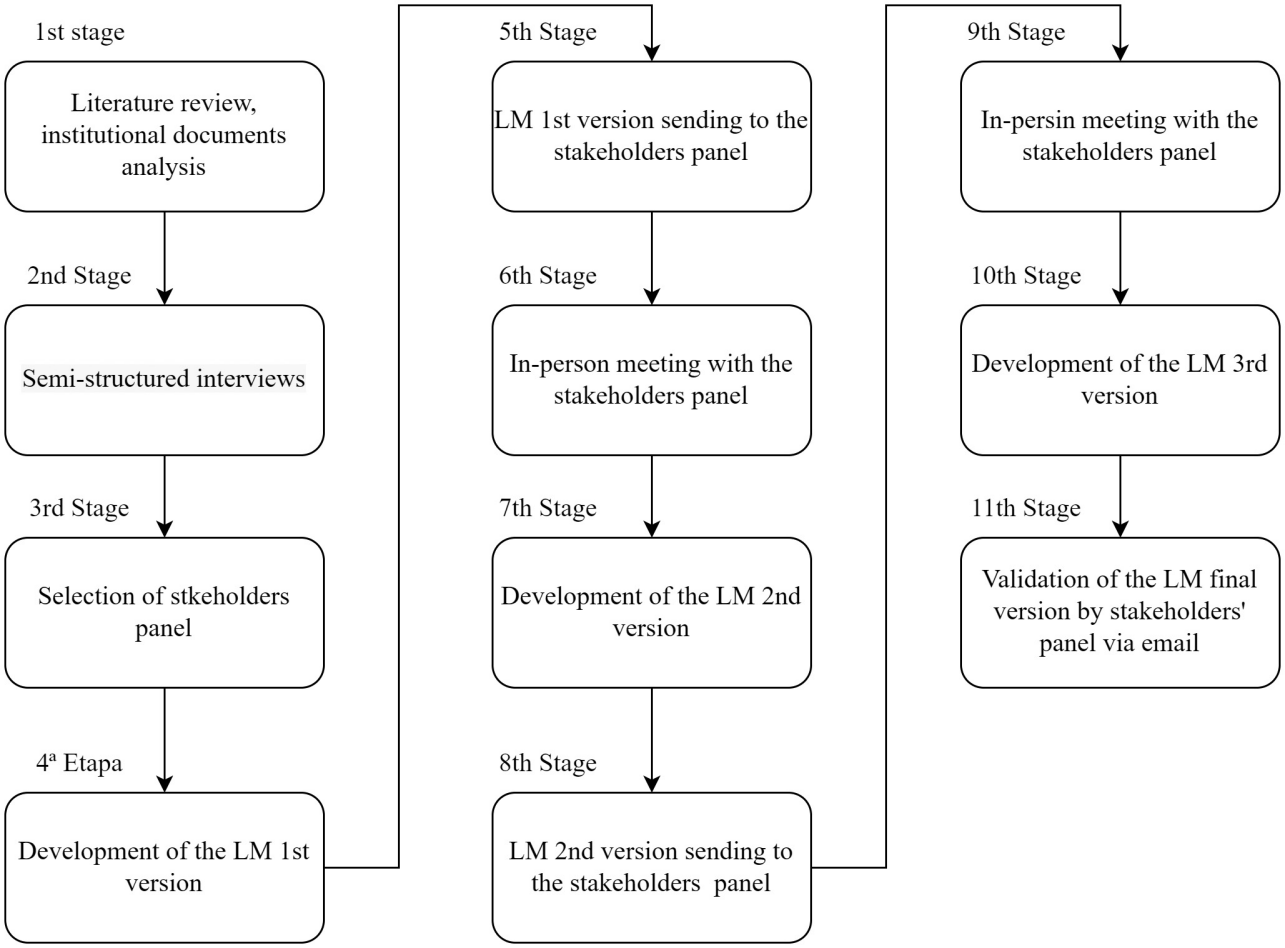
Logical model development steps.

The LM development and validation process took place from July 2020 to October 2021, in 11 stages (figure 1).

### 1^ST^ STAGE

The literature review on the development of LM included articles indexed in national and international databases, technical notes, guides and manuals issued or disseminated from 2004 to 2020^(8,9,10,11,12,13,14,15,16,17,18,19,20,21,22,23)^. The institutional documents related to the program were analyzed, which included the standard operating protocol (SOP) with the fundamentals and details of the activities carried out by the program, the guidelines for the non-pharmacological management of symptoms and the structural models used to record the attendances in the patient’s electronic medical record (EMR).

### 2^ND^ STAGE

Then, semi-structured interviews were carried out in person and individually, with two people in a *PAE* management position, including questions related to the history and elements necessary for the construction of the LM. These interviews were included in the LM development process in this study, as an adaptation due to the pandemic scenario and in the face of the stakeholders’ routine, aiming to optimize and reduce the duration of in-person meetings.

### 3^RD^ STAGE

Five ICESP’s employees were invited to participate in the stakeholders’ panel. The sample definition used, as an inclusion criterion, the selection of employees who work in the *PAE*’s operational sector, coordination, management and direction, with two nurses and three people in *PAE*’s managerial position. All the stakeholders signed the Free and Informed Consent Form (FICF), after the principal investigator had explained the purpose of the study and the steps that would be carried out.

### 4^TH^ STAGE

**Figure 2.**
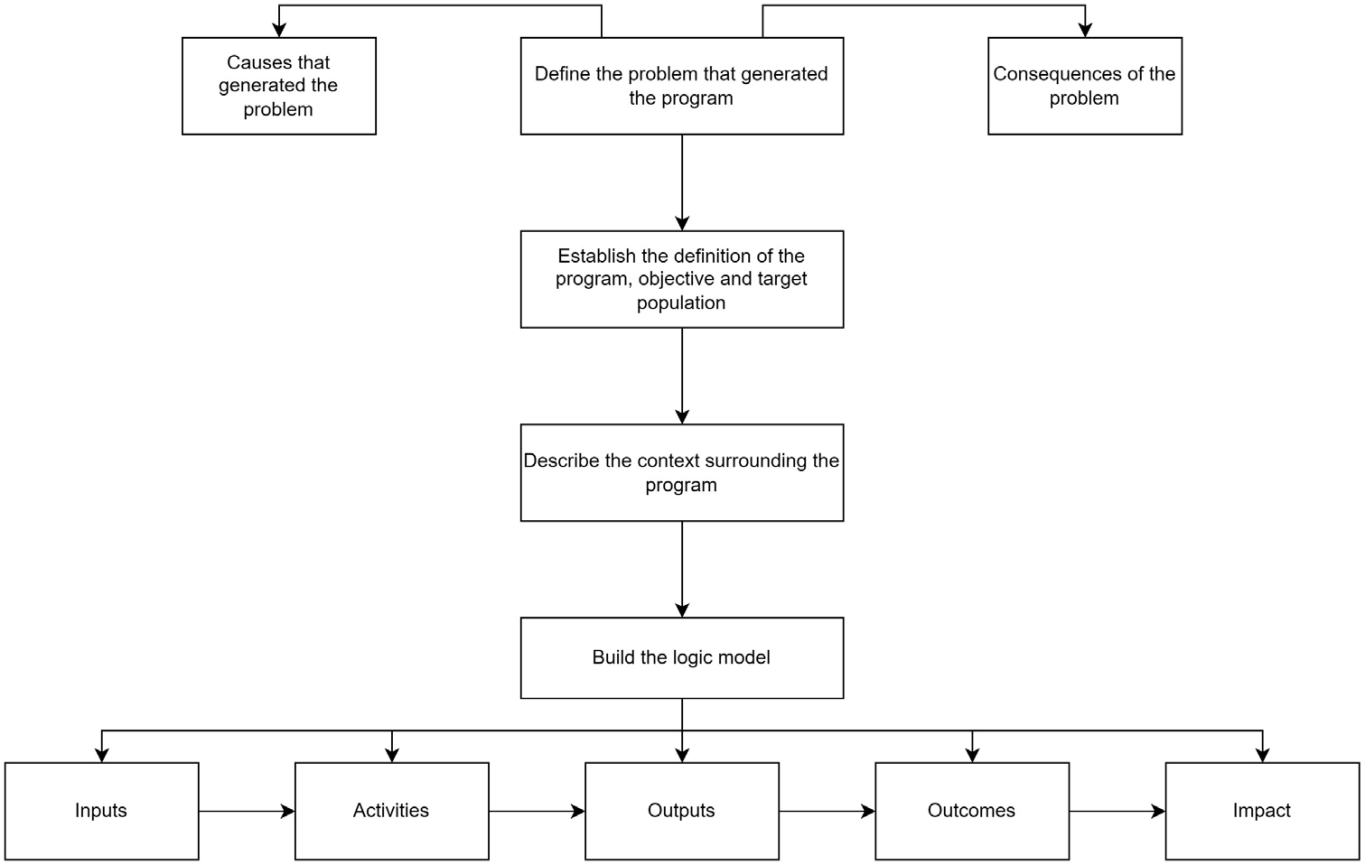
Elements guiding the development of the Logical Model.

Based on the content obtained from the analysis of institutional documents and semi-structured interviews, the first version of the LM was developed, according to the elements described in the flowchart illustrated in Figure 2.

### 5^TH^ STAGE

All the information collected, including the first version of the LM, was written and organized in a didactic material sent by e-mail to the stakeholders five days before the first in-person meeting. In this didactic material, a brief explanatory text was inserted about the concept of LM, its functionality and the definition of stakeholders, so that there was a better understanding of the content, and consequent optimization of the diagram development process. The elements of the LM (definition of the problem, its causes and effects; definition of the *PAE*; general and specific objectives; target audience; the first version of LM containing pillars such as the context in which the *PAE* is inserted, inputs, activities, immediate and intermediate results and impact) were separated by topics with previously collected content, a table with the options “agree”, “partially agree” and “disagree ”, followed by a free space to enter comments. The context was represented by the following components: 1) setting, which describes the environment and conditions in which the program is inserted, 2) epidemiological, specifies the user’s profile regarding the pathology, 3) socioeconomic, which refers to the patient’s socioeconomic conditions, and 4) sociocultural, indicates beliefs, traditions, and habits.

### 6^TH^ STAGE

The first in-person meeting with the stakeholders was held on October 1, 2020, coordinated by the principal investigator, and aimed to discuss the topics of the submitted material and validate the first version of the LM. It lasted approximately 1 hour and 30 minutes. Forty minutes were allotted for the history of the PAE to be retold by the stakeholders who participated in the program’s initial operation, with the objective of helping the others to understand the real reasons that generated the *PAE*, and based on this, the discussion was started to reach consensus on the macro problem. Then, the principal investigator presented the LM elements and visual scheme, previously sent to the stakeholders, and asked the participants to analyze the topics in the material and discuss the conflicting points, until a consensus was reached. The printed didactic material was made available to each of the stakeholders, which they had previously received via e-mail. In this material, following the collective analysis of each topic, the stakeholders were asked to fill in the check box (agree, partially agree or disagree) and the free space for comments. The material filled during the meeting was handed over to the principal investigator, so that the suggestions presented could be included in the LM second version and the diagram redrawn. With all participants’ authorization, the meeting was recorded using audiovisual resources.

### 7^TH^ AND 8^TH^ STAGES

The LM second version was developed, inserted into the didactic material with the same format as the previous one, and sent by email for prior analysis by the participants, seven days before the second in-person meeting with the stakeholders.

### 9^ST^ STAGE

The second in-person meeting with the stakeholders was held in person on May 5, 2021 for the review and validation of the LM second version, which was developed based on the suggestions indicated at the last meeting. The schedule followed the same format as the previous meeting. New suggestions and corrections were recommended in the following elements: consequences of the macro problem, program definition, general objective, specific objective, context (setting, epidemiological, socioeconomic, and sociocultural), inputs, activities, products, results, correlation arrows between activities and products, and between products and results.

### 10^TH^ STAGE

The LM third version was developed from the recommendations and suggestions raised at the second meeting, and signaled in the didactic material made available on the stakeholders panel, which was given to the principal investigator, so that she could count the votes (agree, partially agree, and disagree). For the items voted as “partially agree” or “disagree”, the corrections recommended in the considerations field, for each element, were approved by the stakeholders panel at the time of the meeting and included in the LM third version.

### 11^TH^ STAGE

To minimize possible impacts on the stakeholders’ work routine, LM third and final version was sent via email in a file in PDF format with the content updated according to the recommendations of the second meeting. Information validation took place in the body of the same email sent through standardized response boxes (“agree”, “partially agree” and “disagree”) and a free field intended for considerations for each element of the LM mentioned above.

### Ethical Aspects

The research project was submitted to and approved by the Ethics Committee for the Analysis of Research Projects of the Hospital das Clínicas, Medical School of Universidade de São Paulo (CAPPesq-HCFMUSP), according to resolution 466/2012 of the National Health Council – Ministry of Health. of Health, with Research Protocol No. 4.513.242/2021. The five stakeholders consented to participate in the study after reading and signing the FICF, and a printed copy was delivered to each participant. To preserve the participants’ identity, the letters AE, corresponding to *Alô Enfermeiro*, were used to identify the stakeholders, followed by an Arabic number for each participant: AE1, AE2, AE3, AE4 and AE5.

## RESULTS

The macro problem that generated *PAE* was described as “patients of high complexity in the oncological setting”. Based on this, the causes that turn this patient into a high-complexity individual were identified, and the possible impacts of this context. To reach the definition of these elements, a voting took place in the first in-person meeting with the stakeholders, recorded in the printed teaching material and reviewed by the principal investigator. In this voting, an opposition to the first suggestion of the constructed macro problem was observed, which led to a discussion about the best definition for this element.

**Figure 3.**
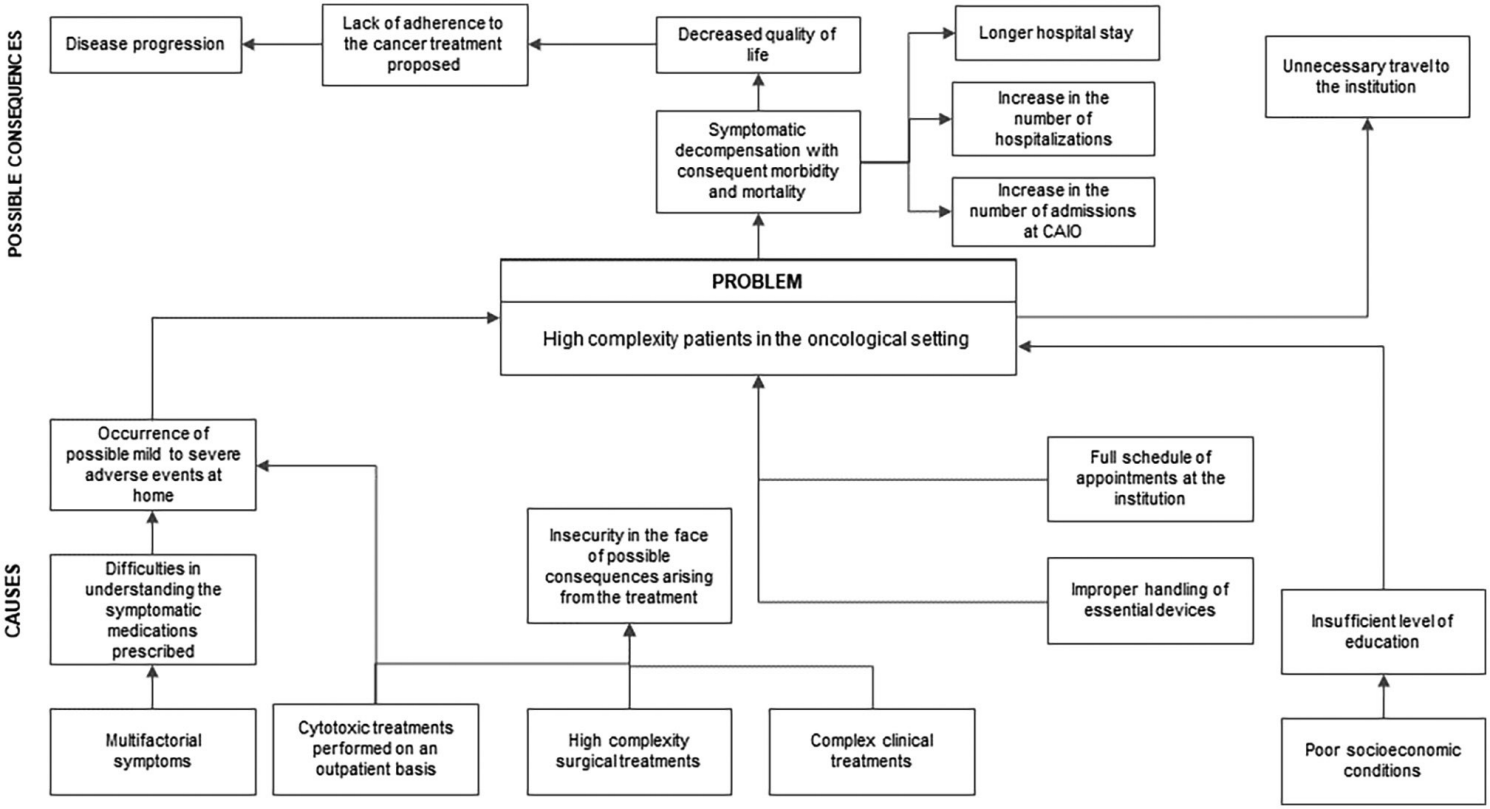
Explanation of the problem generating the PAE.

In the second in-person meeting, with the new definition of the macro problem, two stakeholders proposed the insertion of other consequences that may be related to this macro problem: *increase in the number of hospitalizations (AE2)* and *increase in the number of admissions to the Oncological Complications Care Center (CAIO) (AE1)*. This CAIO is the department dedicated to urgent and emergency care for ICESP’s patients. Identifying “possible consequences” rather than “consequences” was suggested by four stakeholders (AE1, AE3, AE4 and AE5). The stakeholders agreed with the suggested recommendations and recorded the considerations in the didactic material, which was later revised by the principal investigator, who included the changes that generated the third version of the macro problem explanation. This latest version was sent via email to the stakeholders for analysis, and obtained the unanimous vote of “agree” (figure 3).

The following stages in the construction of the LM included the definition of the *PAE*, target audience, general objective and specific objectives:

### Definition of Pae and Target Audience

A TN channel promoting direct communication with the health team professional, in this case the nurse trained for this service, which provides 24-hour daily assistance guidance through a telephone service center, to all patients and/or companions enrolled in ICESP.

### General Objective

To support care self-management and promote continuity of care even outside the hospital environment for outpatients, minimizing hospitalizations resulting from any conditions, and aiming a safe treatment journey through the health team support.

### Specific Objectives

To promote facilitated communication with the health professional, strengthen the bond between the patient/companion and the team, guide behavior directed to the patient’s/companion’s complaint/doubt, support decision-making, reinforce the clinical care management of possible adverse events arising from the therapy or pathology, identify warning signs and clarify doubts pertinent to the treatment journey.

At the second meeting, there were some important statements for the program on the topic “specific objectives”, such as the insertion of “guiding behavior directed at the patient’s/companion’s complaint/doubt, support decision-making”, validated by stakeholders for the next version. One of the stakeholders highlighted the following recommendation relevant to the program’s definition and specific objectives: *To include their companions, as they also make contact with the PAE (AE3)*. This recommendation was reiterated by two other stakeholders (AE4 and AE5) and a consensus was reached in favor of including the words “patient/companion” in the program’s definition elements and specific objectives.

The definition of the PAE, target audience, general objective, and specific objectives was approved by the five stakeholders in the third version sent.

The stage preceding the construction of the diagram consisted of the description of the context in which the PAE is inserted. This was presented at the base of the diagram, with the interaction with the LM being represented by arrows towards the five pillars, which symbolizes that the entire program is inserted within the same context. During the second meeting, four of the five stakeholders partially agreed with the version developed so far. Three stakeholders brought the same suggestions for the socioeconomic and sociocultural topics: precariousness of the basic health network in primary care and religious resistance. The other stakeholders agreed with the insertion of these suggestions.

**Figure 4.**
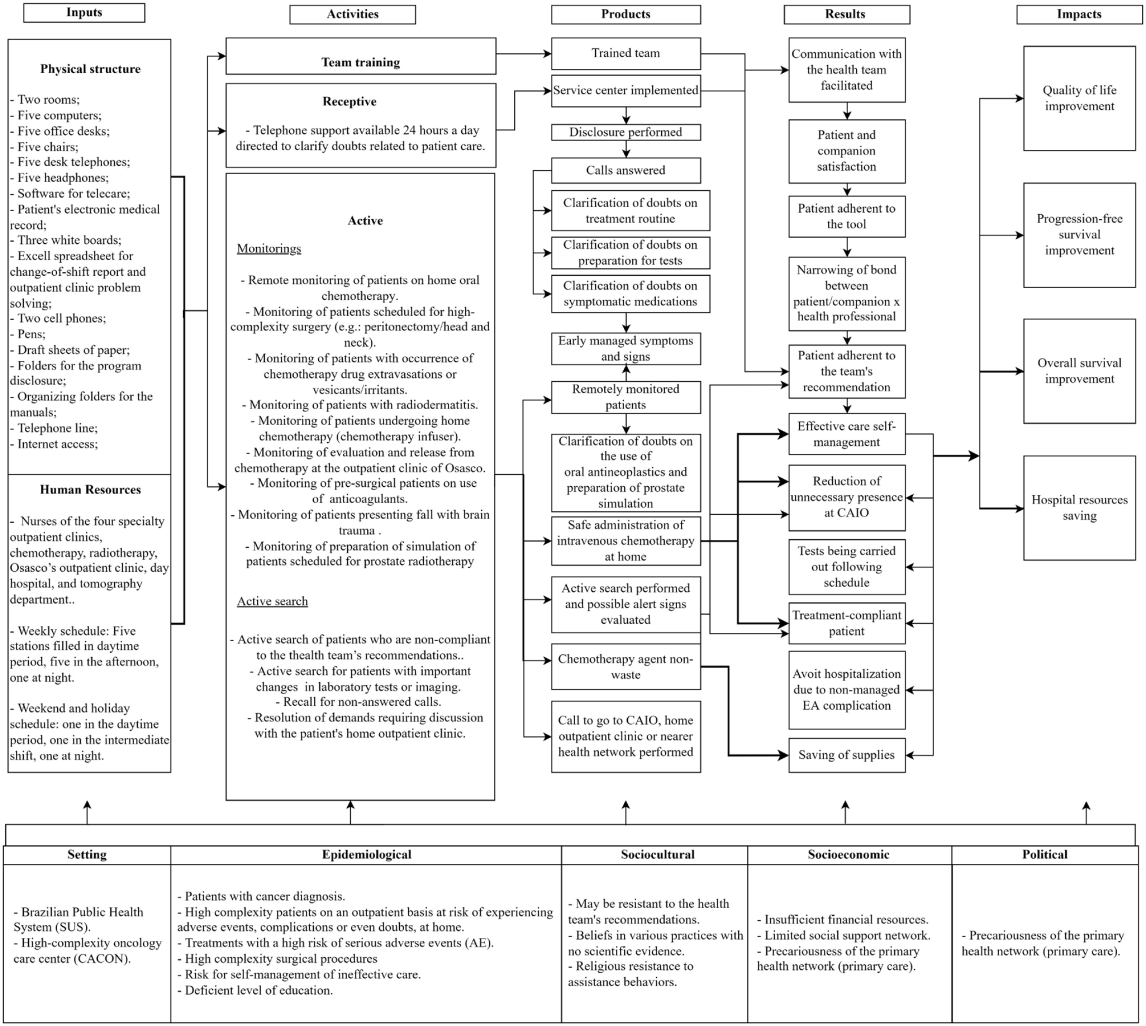
Logical Model (diagram).

The LM diagram was represented by five pillars (figure 4):

Inputs: structure required for program functioning. This pillar was subdivided into physical structure and human resources (nurses, work schedule, and number of positions filled per shift). At the second collective meeting, three stakeholders proposed the use of the current structure of the PAE, which, due to the pandemic scenario and the significant increase in the demands of the program, had the physical and human resources expanded. The other stakeholders agreed with this suggestion, since it represents the structure required for the current *PAE* functioning.

Activities: These are the activities carried out daily in the *PAE*. According to the stakeholders’ suggestion, this pillar was subdivided into receptive team training, when the patient/companion contacts the PAE center, and active team training, when the nurse contacts the patient/companion. In the second in-person meeting, four stakeholders highlighted the importance of including the following activities in the topic “active”: return of unanswered calls and resolution of demands requiring discussion with the patient’s home outpatient’s team. Such recommendations were highlighted in view of the volume of associated demands. In addition to these, two stakeholders (AE1 and AE2) suggested the insertion of another activity in the topic “active”: monitoring the preparation for simulated prostate radiotherapy. The suggested recommendations were approved by the stakeholders’ panel and inserted in the LM third version.

Products: These are short-term results, that is, those achieved with the implementation of activities. At the second collective meeting four stakeholders partially agreed with the version presented, three of these signaling similar considerations: *Include a call to the outpatient clinic or support network (AE3)*.

*Call to the outpatient clinic for evaluation of tubes/probes or, depending on the complaints, emergency room near the patient’s residence (AE4).*

*Call to the ICESP’s outpatient clinic or refer the patient to the health network (AE5).*

The above considerations were presented to the stakeholders panel, who agreed to insert the following description: a call was made to the CAIO, the outpatient clinic of origin or the closest support network.

Still in the pillar “product”, a stakeholder *(AE2)* suggested the insertion of the item: doubts about the use of oral antineoplastic drugs and preparation of a simulated prostate clarified. This item was inserted in view of the cause and effect component arising from the activities: remote monitoring of patients undergoing oral chemotherapy at home and monitoring of preparation for simulated prostate radiotherapy.

Results: These are the medium-term results. In this pillar, the five stakeholders partially agreed during the second collective meeting and signaled the same item: reduction of hospitalization time for unmanaged adverse events (AEs). One of stakeholders emphasized: *I believe that we managed to avoid hospitalization and not to reduce the time (AE5).*

Based on this statement, it was decided that the item in question would be changed to: avoid hospitalization due to an unmanaged AE complication. The consent of the five stakeholders for that item was obtained.

Impacts: These are the long-term results of the PAE. There was unanimous approval of this pillar by the stakeholders during the second collective meeting.

The interaction between the columns, which represents the program complexity, was symbolized by arrows from left to right, which indicated the action and expected effect of each item. In the columns products and results, it was observed that the components inserted in the same pillar could present the phenomenon of cause and effect among themselves, as for example, in the pillar “product” the item “implemented center” has the effect of “disclosure carried out”, which in its turn generates the “answered calls”.

Consensus was reached by the five stakeholders in the third version of the LM, after obtaining the “I agree” vote on all the elements presented.

Based on the LM, it was possible to identify, in fact, the main gap of the PAE, in which evaluation is required. Consensually, the program evaluation should be directed towards the analysis of the results obtained with the implementation, aiming to assess the impact of TN on the admission of patients undergoing chemotherapy to the CAIO.

## DISCUSSION

According to previous studies on program evaluation, the LM is considered one of the best tools to direct and define the evaluative question and the feasibility of the process of a given intervention^(21)^. A TN program developed to optimize the care of patients affected by the SARS-COV2 virus (COVID-19), and to promote agile communication between the family and the health professional, used the LM to support the program evaluation, and as a tool for potential replication in other services^(24)^.

The LM with a theoretical approach contributed to detailing the reason behind the idealization and implementation of the program, promoting the in-depth stakeholders’ analysis of the causes and consequences. In addition, the concept of the program’s basic references, such as definition, target audience, general and specific objective, promoted a consolidated and consensual discourse when describing the PAE.

Studies indicate that starting the construction of the LM based on the macro problem and its respective causes facilitates the process of defining the program objective and activities that will be used to achieve the expected change^(22)^.

Context description is a fundamental element for understanding the factors that can influence the program, acting as a facilitator or barrier for the implementation process and/or achieving results^(23)^. The context in which the PAE is inserted reflects a range of intrinsic and extrinsic challenges to the oncological condition, faced by the patient/caregiver and professionals who are part of the program. Based on the context and its possible impacts on the desired results, it can be observed that the activities making up the PAE were developed and implemented, aiming at the performance of individualized care by a team capable of recognizing the barriers and needs presented by the patient/caregiver. Based on the identified demand, it is possible to establish strategies adapted to the individual’s particularities, aiming at promoting targeted and effective care, seeking to achieve the results outlined in the LM.

In view of the LM structuring, it was possible to understand PAE as a complex intervention in health. This phenomenon is defined from the numerous interactions that exist among the components of an intervention, the amount and level of behaviors required by those who carry out or receive a certain intervention, the organizational levels involved, the different possibilities of results and potential for adaptations^(15)^. The complexity of a telehealth service that provides support to children in rural Australia was based on the development of LM, in addition to allowing a common understanding of the program, and justifying its expansion^(25)^.

The product obtained through the LM construction process consisted of defining the research question aimed at evaluating results, based on the organization of the components that make up PAE, detailing, and meticulous analysis of the expected results. The “reduction in unnecessary attendance at CAIO” was highlighted in the expected results, as it is directly related to the creation of the program, which aims to early manage possible adverse events resulting from the treatment and thus avoid unnecessary admission to the emergency service.

The participation of only five stakeholders, and the absence of a person to represent those who are directly affected by the program, such as the patient and companion, were limiting factors for this study. The absence of the patients’ and companions’ point of view about the PAE limits the understanding of how the program may affect its target audience. Individual values, cultural and socioeconomic factors can affect users’ perception of the program, becoming barriers to the implementation of the *PAE*. Furthermore, the failure to include the expectations, interests, and values of policymakers and program funders may have compromised the description of complexity in the proposed LM. The involvement of multiple stakeholders favors a shared and broader view of what the PAE can do and increases the chance of successful implementation.

After the construction of the PAE LM, other institutions will be able to implement similar programs aiming to promote the continuity of care, quality and safety of care provided to the patient/family, and it is also possible to use the methodology to organize the elements of other interventions, aiming at the success in achieving the results and establishing evaluative questions that are essential for the use of improvements.

## CONCLUSION

The development of the LM allowed the comprehension of the existing structure, and the observation of the interaction between the activities carried out and the expected results of the *PAE*. It is concluded that LM is a functional tool for planning, implementing, and defining evaluative research.
